# Responsiveness and clinically important differences of the PROMIS short form—depression 8a, anxiety 8a, and PASS-20 in individuals with chronic low back pain

**DOI:** 10.1097/PR9.0000000000001170

**Published:** 2024-06-17

**Authors:** Nuttapong Phongsaphakjarukorn, Rotsalai Kanlayanaphotporn, Mark P. Jensen, Prawit Janwantanakul

**Affiliations:** a Department of Physical Therapy, Faculty of Allied Health Sciences, Chulalongkorn University, Bangkok, Thailand; b Department of Rehabilitation Medicine, University of Washington School of Medicine, Seattle, WA, USA

**Keywords:** Anxiety, Depression, Clinically important difference, PROMIS, Responsiveness

## Abstract

**Introduction:**

Depression and anxiety are the most commonly reported mental health conditions. The Patient-Reported Outcomes Measurement Information System Short Form v1.0—Depression 8a (PROMIS-D-8a), Anxiety 8a (PROMIS-Anx8a), and Pain Anxiety Symptoms Scale-20 (PASS-20) measures were designed to assess depression, general anxiety, and pain-related anxiety, respectively.

**Objectives:**

To examine the responsiveness and estimate the clinically important differences (CIDs) of the Thai version of these measures in individuals with chronic low back pain (CLBP).

**Methods:**

The study sample comprised 144 participants with CLBP. Responsiveness was evaluated by calculating the change scores, effect sizes (ESs), standardized response means (SRMs), area under the curve (AUC), and correlations between the change scores and associated Global Perceived Effect (GPE). We also estimated CIDs by the difference in mean change score between improved and unchanged groups and standard error of measurement (SEM) for each measure.

**Results:**

Statistically significant differences in the mean change scores, ESs, and SRMs supported the responsiveness of all measures. The AUCs achieved acceptable discriminatory ability (0.71–0.72) for *moderate* improvement but not for *any* improvement (0.65–0.68). The correlations between GPE and change scores on all measures were low (*r* ranging 0.28–0.33). The estimated CIDs for the PROMIS-D-8a, PROMIS-Anx8a, and PASS-20 were 3.64, 4.20, and 8.80, respectively.

**Conclusion:**

The PROMIS-D-8a, PROMIS-Anx8a, and PASS-20 measures were sensitive for detecting clinical changes over time in individuals with CLBP. The CID values can be used as reference points for assessing meaningful improvements in the domains assessed by these scales in clinical and research practice.

## Abstract 2

The findings supported the responsiveness of PROMIS-D-8a, PROMIS-Anx8a, and PASS-20 in individuals with chronic low back pain, with estimates of the clinically important differences of these measures.

## 1. Introduction

Chronic low back pain (CLBP) is a common condition and a leading cause of global disability.^21,22,34^ Treatments targeting only biomechanical impairments have been recognized as inadequate for effectively managing CLBP.^31,39^ Given that psychological and social components also play a significant role in the experience and impact of CLBP, adequate treatment should also include interventions that address psychosocial issues.^18^ The coexistence of depression and anxiety further complicates the condition.^2,32^ Among individuals with CLBP, the prevalence of depression ranges from 20% to 25%,^23,40,47^ while the prevalence of anxiety ranges from 19% to 24%.^24,47^ An interplay between depression and anxiety has been found to be associated with detrimental outcomes, including increased pain severity,^35,37^ increased disability,^3,35^ and decreased health-related quality of life.^3,41^ Consequently, the presence of significant depression and/or anxiety may serve as barriers to adherence to treatment programs, which can contribute to poor clinical outcomes and an increase in health care costs.^7,25^

Numerous measures are available for assessing depression and anxiety in clinical and research settings.^43,52^ For assessing these domains in individuals with chronic pain conditions, the Patient-Reported Outcomes Measurement Information System (PROMIS) Depression and Anxiety item banks^9^ and the Pain Anxiety Symptoms Scale (PASS)^36^ have been recommended.^5,10^ The PROMIS measures were developed using contemporary psychometric methods (ie, item response theory), which allows for the computation of scores that enable researchers and clinicians to directly compare scores within or across patient groups and different health conditions.^1,9^ Although PROMIS measures can be administered using Computer Adapted Testing (CAT), the static 8-item short form versions (eg, PROMIS SF v1.0—Depression 8a and PROMIS SF v1.0—Anxiety 8a) have been recommended when CAT is not feasible. The PASS was designed to assess pain-related anxiety in individuals with chronic pain.^5^ For use in situations where assessment burden is a concern, a brief 20-item version of the PASS (PASS-20) has been developed.^35^

For measures to be clinically useful, they need to be responsive to change. Estimates of the clinically important differences (CID) in these scales are important for promoting shared decision-making between clinicians and patients in the clinical setting and for classifying research participants as responders or nonresponders in clinical trials.^38,53^ To our knowledge, research to evaluate responsivity and to estimate the CID for the PROMIS SF v1.0—Depression 8a,^29,30^ Anxiety 8a,^28^ and PASS-20 is extremely limited, especially for individuals with chronic pain.

The aim of the current study was to address these knowledge gaps. Specifically, we sought to (1) evaluate the responsiveness to change over time and (2) estimate the CID for the Thai versions of PROMIS SF v1.0—Depression 8a (PROMIS-D-8a), Anxiety 8a (PROMIS-Anx8a), and PASS-20 (PASS-20) in a sample of individuals with CLBP. We hypothesized that these 3 measures would be responsive to change over time in individuals with CLBP, and that the differences in mean changes in the scale scores across the meaningful change groups (ie, improved, unchanged, worsened) would be significantly different.

## 2. Methods

### 2.1. Participants

This study was part of a research project that involved cross-cultural adaptation of the PROMIS-D-8a, PROMIS-Anx8a, and PASS-20 and studied their psychometric properties in individuals with CLBP. The study participants were individuals with CLBP who were identified by physicians or physical therapists as requiring physical therapy treatment. Eligible participants comprised 144 from 4 public hospitals and 5 physical therapy clinics in the Bangkok metropolitan region and nearby provinces. In order to participate in the study, potential participants had to meet the following inclusion criteria: (1) be ≥18 year old; (2) have CLBP, as defined as experiencing pain in the region between the lower posterior margin of the rib cage and the horizontal gluteal fold and which had persisted at least 3 months with pain on at least half the days in the past 6 months^16^; and (3) report an average low back pain intensity in the past 7 days as being ≥3 on a 0 to 10 Numerical Rating Scale (NRS). Participants were excluded if they had a history of severe pathology in the lumbar spine or had a serious medical condition that limited their ability to participate.

### 2.2. Measures

#### 2.2.1. Participant descriptive measures

Participants provided information about their demographics, pain duration, pain intensity, and back-related disability. Current pain and average pain over the past 7 days were measured using the Thai version of the 11-point NRS, ranging from 0 (“No pain”) to 10 (“The worst pain imaginable”).^4^ Back-related disability was assessed using the 10-item Functional Rating Index (FRI) that inquired about specific domains of pain or function (eg, pain intensity, sleep, personal care). Respondents to the FRI rated the severity of pain or disability on 0 (eg, “No pain” or “Perfect sleep”) to 4 (“Worst possible pain” or “Totally disturbed sleep”) Likert scales. Higher scores on the FRI indicate more pain-related disability.^17^ The Thai version of the FRI has been shown to be reliable and valid in the Thai patients with low back pain with the internal consistency (Cronbach alpha) of 0.86.^11^ The internal consistency of the FRI as measured by the Cronbach alpha in the current sample was 0.86, indicating a good reliability.

#### 2.2.2. General depression and anxiety

Depression and anxiety were assessed using the PROMIS SF v1.0 Depression 8a and Anxiety 8a static scales, respectively. With these scales, respondents rate the frequency that they experience specific depression or anxiety symptoms in the past 7 days using a 1 (“Never”) to 5 (“Always”) scale. Responses were summed and then converted to T-scores using conversion tables, which have a mean of 50 and SD of 10 for the U.S. general population.^8^ As with all PROMIS measures, higher T-scores indicate more of the domain being assessed; here, more depression or anxiety. T-scores were categorized as indicating the following severity levels of depression and anxiety: 55 to 59 for mild, 60 to 69 for moderate, and ≥ 70 for severe (see http://www.healthmeasures.net). Previous studies have shown the English versions of these scales to be reliable and valid.^8,20,28,48^ Similarly, the Thai versions of both measures have been shown to be reliable and valid in a sample of CLBP with the internal consistency (Cronbach alphas) of 0.94 for the Thai versions of PROMIS-D-8a and 0.95 for PROMIS-Anx8a.^12,44^ The internal consistency reliability (Cronbach alphas) for these measures in the current sample were 0.91 and 0.94, respectively, indicating excellent reliability.

#### 2.2.3. Pain-related anxiety

The 20-item brief version of the Pain Anxiety Symptoms Scale (PASS-20) was used to assess pain-related anxiety. The PASS-20 measures 4 domains of pain-related fear: fear of pain, cognitive anxiety, physiological anxiety, and behavioral (ie, escape/avoidance) aspects of fear.^35^ Respondents to the PASS-20 rate the frequency that they experience the fear response described by each item on a 0 (“Never”) to 6 (“Always”) scale. Responses are summed to compute each scale score, and the scores for each can range from 0 to 25; the total score can range from 0 to 100. Higher scores indicate more anxiety. Respondents can be classified into one of 3 groups based on their total score: mild (0–34), moderate (34–67), and severe (68–100).^6^ The original English version of the PASS-20 has been shown to be reliable and valid in chronic pain populations.^14,35^ The total scale score of the Thai version of the PASS-20 has also been shown to be reliable and valid in a sample of CLBP with the internal consistency (Cronbach alpha) of 0.95.^12^ The internal consistency (Cronbach alpha) of the PASS-20 in the current sample was 0.94, indicating excellent reliability.

#### 2.2.4. Global perceived effect

Two 7-point Global Perceived Effect (GPE) scales (one each assessing perceived global change in depression and the other assessing global perceived change in anxiety) were administered after 4 weeks of physical therapy treatment. The GPE asks respondents to rate the change in their depression/anxiety symptoms since the initial assessment. Respondents indicated the level of improvement or worsening in the specified symptom on 1 to 7 scales (1 = “Very much improved,” 2 = “Much improved,” 3 = “Slightly improved,” 4 = “Not changed,” 5 = “Slightly worsened,” 6 = “Much worsened,” and 7 = “Very much worsened”). The GPE scale has been shown to be reliable in patients with musculoskeletal conditions.^26^

### 2.3. Procedures

Data collection was conducted from February 2023 to August 2023. Before signing the informed consent form, the study procedures were described to all participants. At the initial assessment, the participants administered the demographic questionnaire, NRS, FRI, as well as the 3 measures of depression and anxiety described above. Four weeks after receiving standard physical therapy treatment (see Results section for a more detailed description of the treatment options provided to the participants as a part of standard care), the participants were again administered the 3 depression and anxiety measures, as well as the GPE. Participants had the option to return their completed questionnaires either in person upon return to the clinic or by mail. Ethical approval of the current study was provided by the Research Ethics Review Committee for Research Involving Human Research Participants, Group I, Chulalongkorn University (COA No. 208/65).

### 2.4. Data analysis

Descriptive statistics were computed to describe the sample and study variables. We next determined the possible floor and ceiling effects by computing the percentage of participants scoring the lowest and highest possible scores for each measure. We determined a priori that a measure would have a significant risk for floor or ceiling effects, if the percentage of lowest or highest possible scores was >15%.^49^

We evaluated responsiveness based on distribution- and anchor‐based methods.^33,42^ For the distribution-based method, the effect size (ES) and standardized response mean (SRM) were calculated. Based on their response to the GPE scale, participants were categorized twice (one for change in depression and the other for change in anxiety) into 3 groups for the intended analyses: improved (1–3), unchanged (4), and worsened (5–7). Change scores in all study measures over time were calculated by subtracting the initial (pre physical therapy) score from 4-week follow-up scores; a positive value indicated a worsening while a negative value indicated an improvement. The ES was estimated by measuring Cohen *d*, which was computed by dividing mean change score by the SD of baseline scores. The SRM was calculated by dividing mean change score by the SD of that change scores.^13^ For both ES and SRM, values of 0.2, 0.5, and 0.8 were deemed to represent small, moderate, and large responsiveness, respectively.^27^

For the anchor-based method, the change scores for each measure for the 3 categorized groups (ie, improved, unchanged, worsened) were compared using the Kruskal-Wallis test. The area under the curve (AUC) was used to examine the discriminate ability of the scales between participants who reported that they improved and those who reported that they did not improve.^49^ Two levels of improvement, which were *any improvement* (GPE score of 1–3) and *moderate improvement* (GPE score of 1–2), were studied.^30^ Those with scores of 4 to 7 were considered not improved. An AUC of at least 0.70 was deemed acceptable.^49^ The Spearman rank order correlation coefficients (*r*) between the change scores for all measures and the associated GPE score at 4-week assessment were calculated. The correlation of at least 0.30 was deemed acceptable.^42^

Finally, we utilized anchor-based and distribution-based methods to calculate the CID.^42^ The anchor-based CID was calculated using the difference in mean change score between improved and unchanged groups.^15,53^ This value estimates change in the outcome measure score that is linked to a meaningful external anchor from the patient's perspective.^53^ The distribution-based CID was calculated using the standard error of measurement (SEM, by using the SD of baseline scores multiplied by square root of 1 − ICC_(2,1)_). The SEM indicates the precision of the outcome measure and can be interpreted as the smallest amount of change that is likely to reflect true change.^42^ The ICC values for all study measures were computed using data from participants who reported their condition in each domain as being “not changed” at the 4-week follow-up. A suitable anchor-based CID should exceed the measurement error.^42^ All statistical analyses were conducted using SPSS version 29.0. A *P*-value *<* 0.05 was considered statistically significant.

## 3. Results

### 3.1. Participants and treatment

Of 287 individuals initially approached, 252 (88%) expressed interest in participation. Of these, 167 met the study's inclusion and exclusion criteria. Twenty-three participants did not provide data at the second assessment point; 19 were lost to follow-up and 4 withdrew. During the 4-week study period, all participants received one or more standard physical therapy treatments, which were tailored to their individual needs by their physical therapists (eg, manual therapy, lumbar traction, ultrasound therapy, electrical stimulation for pain management, core stabilization exercise, or massage). No specific physical therapy treatment recommendation (including treatments that should be provided or avoided) was made or required for an individual to participate in the current study. Similarly, no limitation was placed on receiving any other pharmacological or nonpharmacological treatments for CLBP. The demographic data of the 144 study participants are presented in Table 1. Notably, participants reported having a moderate level of pain intensity on the NRS and moderate disability on the FRI.

**Table 1:** Demographic data of the participants (n = 144).

Variables	n (%)	Mean (SD)
Sex		
Female	102 (71)	
Male	42 (29)	
Age (y)		52.4 (15.9)
Height (cm)		160.3 (8.7)
Weight (kg)		65.3 (13.9)
Body mass index (kg/m^2^)		25.3 (4.6)
Pain duration (mo)		39.7 (41.1)
Pain intensity (NRS; 0–10)		
Current pain		5.8 (1.6)
Average pain (7-d)		5.8 (1.5)
Disability (FRI; 0–100)		47.5 (14.7)

FRI, functional rating index; NRS, numeric rating scale; SD, standard deviation.

Table 2 presents the means and SDs of the initial and follow-up scores of the study measures, as well as the percentage of participants who scored at the highest and lowest possible levels for each measure. On average, participants reported little or no depressive symptoms on the PROMIS-D-8a (mean = 52.7), mild anxiety on PROMIS-Anx8a (mean = 57.0), and moderate pain-related anxiety on PASS-20 (mean = 44.9). A risk for the floor effect was found only for the PROMIS-D-8a, with a 16% occurrence at baseline and a somewhat higher risk at the 4-week follow-up.

**Table 2:** Mean baseline (SD), mean 4-week follow-up scores (SD), and percent of participants who provided the lowest and highest possible scores on the patient-reported outcomes measurement information system short form—depression 8a, patient-reported outcomes measurement information system short form—anxiety 8a, and pain anxiety symptoms scale-20 (n = 144).

	Mean (SD)		Percent of response at baseline		Percent of response at 4-wk	
	Baseline	4-wk	Lowest score	Highest score	Lowest score	Highest score
PROMIS-D-8a	52.7 (8.6)	51.3 (8.2)	16	0	19	0
PROMIS-Anx8a	57.0 (9.6)	54.0 (8.8)	10	1	11	1
PASS-20	44.9 (21.2)	38.5 (19.6)	1	0	1	0

PROMIS-D-8a, patient-reported outcomes measurement information system short form—depression 8a; PROMIS-Anx8a, patient-reported outcomes measurement information system short form—anxiety 8a; PASS-20, pain anxiety symptoms scale-20; SD, standard deviation.

### 3.2. Responsiveness

Table 3 summarizes the mean change scores with SD, ESs, and SRMs for all measures in the improved and unchanged groups. As only 4 participants reported worsened depression and anxiety over the 4 weeks of treatment, the mean change scores, ESs, and SRMs were not computed in these groups. Therefore, comparisons were only made between the improved and unchanged groups using the Mann-Whitney *U* test instead of the initially planned Kruskal-Wallis test. The results demonstrated statistically significant differences in change scores between the improved and unchanged groups in all measures (*P* values = 0.003 for PROMIS-D-8a, 0.005 for PROMIS-Anx8a, and 0.028 for PASS-20). Within the improved group, all 3 measured exhibited improvement with negative change scores. The absolute values for the ESs and SRMs for each measure ranged from 0.30 to 0.46. For the unchanged group, the absolute values of the ESs and SRMs were less than 0.20 for all measures.

**Table 3:** Mean change scores (SD), effect sizes, and standardized response means of the patient-reported outcomes measurement information system short form—depression 8a, patient-reported outcomes measurement information system short form—anxiety 8a, and pain anxiety symptoms scale-20.

	Improved, n		Unchanged, n		*P* *
Mean change scores from baseline (SD)					
PROMIS-D-8a	−2.39 (5.22)	100	0.15 (5.73)	40	0.003
PROMIS-Anx8a	−3.68 (8.45)	115	0.52 (5.03)	25	0.005
PASS-20	−8.08 (17.47)	115	−0.72 (7.61)	25	0.028
Effect sizes					
PROMIS-D-8a	−0.30	100	0.02	40	
PROMIS-Anx8a	−0.41	115	0.05	25	
PASS-20	−0.40	115	−0.03	25	
Standardized response mean					
PROMIS-D-8a	−0.46	100	0.03	40	
PROMIS-Anx8a	−0.44	115	0.10	25	
PASS-20	−0.46	115	−0.09	25	

The worsened group was not included due to very few participants (n = 4 in depression and anxiety).

* Mann-Whitney *U* test was used to compare improved and unchanged group.

PROMIS-D-8a, patient-reported outcomes measurement information system short form—depression 8a; PROMIS-Anx8a, patient-reported outcomes measurement information system short form—anxiety 8a; PASS-20, pain anxiety symptoms scale-20; SD, standard deviation.

The results from AUC analysis indicated acceptable discriminate ability for the 3 study measures for detecting *moderate* improvement (AUC ranges: 0.71–0.72) (Table 4). However, the ability to detect *any* improvement, with AUC values ranging from 0.65 to 0.68, fell slightly below the acceptable threshold. The correlations between change scores and the corresponding GPE ratings at 4-week assessment point were acceptable for PROMIS-Anx8a and PASS-20 (*r* ≥ 0.30), but it was lower than expected (*r* < 0.30) for PROMIS-D-8a.

**Table 4:** Area under the curve and correlations coefficients of the patient-reported outcomes measurement information system short form—depression 8a, patient-reported outcomes measurement information system short form—anxiety 8a, and pain anxiety symptoms scale-20 with global perceived effect in individuals with chronic low back pain.

	AUC (95% CI)		Correlation with GPE	
Measure	Any improvement	Moderate improvement	*r*	*P*
PROMIS-D-8a	0.68 (0.58–0.77)	0.72 (0.62–0.81)	0.28	<0.001
PROMIS-Anx8a	0.66 (0.56–0.76)	0.72 (0.61–0.82)	0.31	<0.001
PASS-20	0.65 (0.55–0.74)	0.71 (0.62–0.81)	0.33	<0.001

AUC, area under the curve; CI, confidence interval; GPE, global perceived effect; PASS-20, pain anxiety symptoms scale-20; PROMIS-Anx8a, patient-reported outcomes measurement information system short form—anxiety 8a; PROMIS-D-8a, patient-reported outcomes measurement information system short form—depression 8a.

### 3.3. Clinically important difference estimates

Over the 4 weeks of treatment, 40 participants reported no change in depression and 25 reported no change in anxiety. ICC values were 0.82 for PROMIS-D-8a, 0.87 for PROMIS-Anx8a, and 0.93 for PASS-20. Using the distribution-based estimates, the SEM was 3.64 for PROMIS-D-8a, 3.43 for PROMIS-Anx8a, and 5.61 for PASS-20. Anchor-based estimates yielded absolute value of the CID at 2.54, 4.20, and 8.80 for PROMIS-D8a, PROMIS-Anx8a, and PASS-20, respectively (Table 5). Notably, only the anchor-based CID estimate for PROMIS-D-8a was lower than the SEM. Therefore, the final CID estimates were 3.64 for PROMIS-D-8a, 4.20 for PROMIS-Anx8a, and 8.80 for PASS-20.

**Table 5:** Clinically important difference calculated as between-group mean change score (improved vs unchanged) at 4 weeks and standard error of measurement for the patient-reported outcomes measurement information system short form—depression 8a, patient-reported outcomes measurement information system short form—anxiety 8a, and pain anxiety symptoms scale-20.

Measure	Between-group mean change score	SEM
PROMIS-D-8a	2.54	**3.64**
PROMIS-Anx8a	**4.20**	3.43
PASS-20	**8.80**	5.61

The estimated CID chosen is in bold face text.

CID, clinically important difference; SEM, standard error of measurement.

## 4. Discussion

This prospective study describes the evaluation of responsiveness and CID of Thai versions of the PROMIS-D-8a, PROMIS-Anx8a, and PASS-20 in a sample of individuals with CLBP who were treated by physical therapists. The changes observed in the study participants are considered clinically important at the group level. The findings have important implications for how these measures might be used in clinical and research settings.

### 4.1. Responsiveness

Most of the study findings supported the responsiveness of 3 measures. The current study demonstrated that all study measures had the small to moderate responsiveness in the improved group. However, previous studies reported moderate effect size for the English version of PROMIS SF v1.0 Depression 8a (SRM = 0.49 in an improved group) in a sample of individuals with CLBP and the PROMIS SF v1.0 Anxiety 8a (SRM = 0.56 in an improved group) in a sample of individuals with chronic musculoskeletal conditions.^28,30^ This inconsistency might be due to the fact that the current study participants received standard physical therapy as the primary intervention, which does not specifically target the constructs measured (ie, depression and anxiety). In contrast, previous studies that reported moderate effect sizes have utilized interventions that were more directly aligned with the psychological constructs being assessed. In addition, the follow-up periods in the previous studies were longer (3 and 6 months) compared to the 4-week follow-up period of the current study. These different follow-up periods may have allowed participants to experience greater improvement due to longer treatment courses or the natural course of symptom recovery.

Regarding the PASS-20, no previous studies have investigated its responsiveness. Nevertheless, we found that the PASS-20 demonstrated comparable responsiveness to change to the PROMIS-Anx8a in the improved group. However, we were unable to evaluate the responsiveness of all measures for detecting *worsening* in depression and anxiety as the number of participants reported worse outcomes in the current sample was too low to be able to compute reliable estimates for any measure.

The findings indicated that all 3 measures had acceptable ability to discriminate between improved and unimproved participants for detecting *moderate improvement*. However, their ability to discriminate was lower when detecting *any improvement*. The AUC values for PROMIS-Anx8a and PROMIS-D-8a in this study were somewhat higher than those reported in previous research of the English version (AUC = 0.63 for PROMIS SF v1.0 Depression 8a and AUC = 0.65 for PROMIS SF v1.0 Anxiety 8a).^28,30^ This inconsistency may be attributed to the shorter follow-up period in the current study (1 month) compared to previous research (3 months and 6 months). With longer follow-up period, it is possible that participants may not accurately recall their baseline symptom states for comparison, or a greater number of individuals may start to return to baseline levels.^19,26,45^ Given that the PASS-20 has not yet been evaluated for its ability to discriminate individuals who have improved or not improved in anxiety, the current findings on this issue are novel and cannot be directly compared with prior research.

### 4.2. Clinically important difference estimates

The CID estimates are consistent with what has been reported in other studies for the PROMIS measures, which also suggested the optimal minimal CID estimates of PROMIS SF v1.0 Depression 8a between 3 and 4 points and about 4 points for PROMIS SF v1.0 Anxiety 8a.^28,29^ The CID values estimated in this study can be utilized as benchmarks for assessing the magnitude of improvement in the PROMIS SF v1.0 Depression 8a, PROMIS SF v1.0 Anxiety 8a, and PASS-20 scores, reflecting a numerical threshold of reliable change. Apart from evaluating treatment effectiveness, CID plays a significant role in sample size calculation for research studies to ensure adequate statistical power and to minimize false positive and negative conclusions.^51,53^ Clinicians and researchers should recognize that these estimated CID values were derived from a group of participants and may not necessarily reflect the change needed to determine that the change observed is meaningful for an individual. Instead, in the clinical context, the CID can be used as an indication of the probability of meaningful change, rather than serving as an absolute indicator of treatment success.^46,50^ By considering the CID, clinicians and researchers might better understand patient responses to treatment, tailor interventions to meet patient needs, and enhance the quality of care provided. However, it must be noted that estimates of the CID can be influenced by the study population and technique used; further research to evaluate the reliability of the current findings is warranted.

### 4.3. Limitations

The study has several limitations that should be considered when interpreting the findings. First, the study sample consisted of individuals with CLBP. The extent to which they generalize to samples of individuals with other chronic musculoskeletal pain conditions is not known. Further research in diverse populations is needed to evaluate the reliability of these findings across different populations with chronic pain. Second, although the findings provide clearly defined responsiveness estimates for the measures studied in the context of improvement, we were unable to evaluate the ability of the measures to detect worsening in depression and anxiety over time, due to the very small number of participants who reported that they became worse over time. This again supports the need to evaluate these measures in additional samples of individuals with chronic pain; for example, in individuals who recently completed treatment focusing on improving psychological function, a subset of whom might be expected to deteriorate over time after treatment. Finally, the accuracy of CID estimation based on between-group differences is heavily influenced by the accuracy of the anchors used.^42,50^ The low correlations between the study measures and the anchor may incur more bias compared to other anchor-based methods.

## 5. Summary and conclusions

Despite the study's limitations, the findings provide important new information regarding the psychometric properties of 3 important measures of psychological function in a sample of individuals with chronic pain. Our study provides strong evidence for detecting change over time in the construct being measured. Moreover, incorporating these CIDs into treatment planning can help clinicians or researchers to assess probability that changes in depression or anxiety in individuals with CLBP represent a meaningful improvement.

## Disclosures

This study was funded by the 90th Anniversary of Chulalongkorn University Fund (GCUGR1125661055M). A scholarship from the Graduate School, Chulalongkorn University to commemorate the 72th anniversary of his Majesty King Bhumibol Adulyadej is gratefully acknowledged.
